# Heart failure with supranormal ejection fraction: clinical characteristics and outcomes compared to mildly reduced and preserved ejection fraction

**DOI:** 10.1007/s00392-025-02620-9

**Published:** 2025-02-24

**Authors:** Amitai Segev, Rotem Tal-Ben Ishay, Marco Metra, Elad Maor, Dov Freimark, Anan Younis, Roy Beigel, Shlomi Matetzky, Avishay Grupper

**Affiliations:** 1 Cardiovascular Division, The Leviev Heart Center, Sheba Medical Center, Tel-HaShomer, Sheba Rd. 2, Ramat-Gan, Israel; 2 Faculty of Medicine, Tel Aviv University, Tel Aviv, Israel; 3 Department of Medical and Surgical Specialties, Radiological Sciences and Public Health, Institute of Cardiology, ASST Spedali Civili, University of Brescia, Brescia, Italy

**Keywords:** HFsnEF, Supranormal EF, Prognosis, Characteristics, Outcomes

## Abstract

**Background:**

Little is known about the recently emerging entity, heart failure with supranormal ejection fraction (HFsnEF).

**Objective:**

To describe the clinical characteristics and outcome of HFsnEF, compared to HF with mildly reduced EF (HFmrEF) and HF with preserved EF (HFpEF) patients.

**Design:**

A single center retrospective analysis.

**Patients:**

Hospitalized and ambulatory heart failure (HF) patients who underwent echocardiography with left ventricular ejection fraction (LVEF) > 40%.

**Main measures:**

Clinical and echocardiographic parameters, hospitalization rates and mortality.

**Key results:**

A total of 6,202 patients (mean age 81.4 ± 14.1 years, 52% females) were analyzed: 750 in the HFmrEF group (LVEF 41–49%), 4360 in the HFpEF group (LVEF 50–64%), and 1092 in the HFsnEF group (LVEF ≥ 65%). Patients were followed for a median of 32 (11–65) months. HFsnEF patients were older, predominantly female, exhibited higher hypertension prevalence, more severe LV hypertrophy, smaller LV dimensions, and higher filling pressures compared to the other groups (p < 0.001 for all). These features were consistent in both hospitalized and ambulatory patients. In a univariable model, HFsnEF patients had higher mortality rates compared to HFmrEF and HFpEF patients (HR 1.258, 95% CI 1.117–1.418; p < 0.001 and HR 1.112, 95% CI 1.023–1.208; p = 0.012, respectively). However, in a multivariable model, adjusted for age, sex, comorbidities, and echocardiographic parameters, there was no significant difference in the mortality rates between all groups. The total hospitalization rate was similar between the HFpEF and HFsnEF groups, and lower in the HFmrEF group (p = 0.022). However, the HFsnEF group had the lowest rate of HF-related hospitalizations (p = 0.002).

**Conclusion:**

HFsnEF represents a group of patients with a distinct clinical and echocardiographic profile accompanied by worse outcomes, likely mediated by older age and a higher comorbidity burden, compared to HFmrEF and HFpEF. Therefore, the supranormal EF may serve as a marker rather than an independent prognostic factor.

**Graphical abstract:**

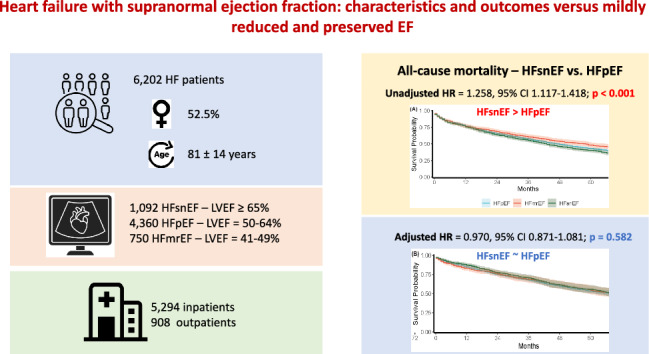

**Supplementary Information:**

The online version contains supplementary material available at 10.1007/s00392-025-02620-9.

## Introduction

Despite recent advances in our understanding of the fundamental mechanisms underlying heart failure (HF), patients with HF are still classified based on their left ventricular ejection fraction (LVEF) due to varying prognosis and treatment response [1, 2].

Prior studies demonstrated the differential effect of HF medications on HF with mildly reduced EF (HFmrEF) versus HF with preserved EF (HFpEF) patients [3–5]. Recently, there has been growing interest in patients with supranormal EF (snEF), with more studies investigating this patient population. Moreover, the only medical treatment that currently demonstrated significant prognostic benefit in patients with HFpEF, sodium-glucose cotransporter 2 inhibitors (SGLT2i) [6–8], have also demonstrated inconsistent results in the snEF range [6–8]. While dapagliflozin demonstrated consistent benefit across the entire spectrum of LVEF [7], the positive effect of empagliflozin was attenuated in patients with LVEF ≥ 65% [8]. These contradictory results emphasize the need to further characterize and understand the underlying mechanisms as well as the prognostic meaning of HF with snEF (HFsnEF).

The first large-scale clinical cohort study to investigate this topic included a heterogeneous population of 203,135 patients who underwent 403,977 echocardiograms over a twenty-year period. This study linked a high LVEF (≥ 65%) to increased mortality and defined this subgroup of HFsnEF patients as an intriguing new phenotype, the underlying mechanisms of which remain unknown [9]. Since then, several studies have investigated the prognosis of patients with snEF and reported a significant association between high LVEF and all-cause mortality in asymptomatic patients with no cardiovascular (CV) disease [10, 11].

Most recently, a sub-analysis of the RELAX-AHF-2 trial has shown comparable all cause and CV mortality rates among all HF groups yet a higher risk of non-CV death in patients with HFsnEF (LVEF > 65%) [11].

These results suggest a U-shaped relationship between LVEF and the risk of adverse outcomes, with an increased risk of mortality in patients with a low LVEF, but also in patients with a snEF. However, these studies examined heterogeneous groups of patients including healthy individuals, used different modalities to estimate LVEF and lacked uniformity in defining snEF cut-off values. Therefore, the characteristics of patients with HFsnEF and the impact of snEF on clinical outcomes within HF patients remain unclear.

Our study aimed to characterize the profile and clinical course of patients with HFsnEF compared to HFmrEF and HFpEF among a large cohort of patients with a diagnosis of HF.

## Methods

### Data collection

We conducted a retrospective single-center cohort study at the Sheba medical center, a tertiary referral hospital, between January 2009 – March 2023. Our cohort comprised two subpopulations: hospitalized patients with a primary discharge diagnosis of HF or related ICD-10 code, and ambulatory patients who received a HF diagnosis at the outpatient CV clinics in our center. The initial cohort consisted of 14,549 inpatients and 4693 outpatients. We included only patients who underwent a formal echocardiographic evaluation at the Sheba medical center echo lab within six months of the outpatient clinic HF diagnosis or index HF hospitalization, resulting in 9400 inpatients and 2801 outpatients. We further excluded 4100 inpatients and 1806 outpatients with reduced LVEF (HFrEF; LVEF ≤ 40%) and 414 duplicates. The final cohort for the statistical analysis was comprised of 6202 patients (5294 inpatients and 908 outpatients). Data was extracted from patients’ electronic medical records using a data curation platform (www.mdclone.com) [12]. Mortality data was extracted from the Israeli National Population Registry and was available for all cases. The data collected included demographic variables (e.g., age and sex), comorbidities (e.g., ischemic heart disease (IHD) and diabetes mellitus), chronic medications, laboratory results (from the first available measurement in the hospitalized cohort and the nearest available exam within 3 months in the outpatient cohort), and echocardiographic parameters. Possible confounders that may contribute to a hyperdynamic left ventricle (LV) were examined in the inpatient cohort: acute anemia (as determined by hemoglobin levels and appropriate diagnoses at discharge), sepsis (based on maximal fever, positive blood and/or urine cultures, and appropriate diagnoses at discharge), cirrhosis, carcinoid, and gastrointestinal bleeding (by appropriate diagnoses during hospitalization). The primary outcome was all-cause mortality. The secondary outcome was time to total and HF-related hospitalizations. Hospitalizations were classified as HF-related if a primary admission or discharge diagnosis of HF or related ICD-10 code was recorded.

The Institutional Review Board of the Sheba medical center approved this study based on strict maintenance of participants’ anonymity during database analyses. No individual consent was obtained. Patients and the Public were not involved in the research design or performance. The data underlying this article will be shared on reasonable request to the corresponding author.

### Statistical analysis

Patients were divided into three groups by their LVEF: HFmrEF (LVEF 41–49%), HFpEF (LVEF 50–64%), and HFsnEF (LVEF ≥ 65%). Baseline characteristics are presented as mean and standard deviation for normally distributed continuous variables, median and inter-quartile range (IQR) for not normally distributed continuous variables and for discrete quantitative variables, and count and percentage for qualitative variables. The differences between the groups were examined using ANOVA and Kruskal–Wallis tests for qualitative variables by their distribution. The chi-square test was used to examine categorical variables, with Linear-by-Linear association when appropriate.

For mortality analysis, a univariable analysis of Kaplan–Meier survival plots was performed with the Log-rank test to compare the three groups. Demographic and clinical variables that were found significantly different between the groups were examined using Kaplan–Meier and univariable COX regression with time-to-death as the dependent variable. Selected variables, based on their clinical and statistical significance, were included in a multivariable COX regression model.

## Results

The study cohort included 6202 patients (mean age 81.4 ± 14.1 years, 52% females); 5294 inpatients (85%), and 908 outpatients (15%), who were categorized into three groups based on their LVEF: HFmrEF group (LVEF ≤ 40%; n = 750), HFpEF group (LVEF 50–64%; n = 4360) and HFsnEF group (LVEF ≥ 65%; n = 1092).

### Clinical characteristics

Baseline characteristics are presented in Table 1. Patients in the HFsnEF group were older, more commonly females, and had higher rates of hypertension compared to the HFmrEF and HFpEF groups (p < 0.001 for all). Baseline laboratory parameters revealed slightly lower hemoglobin and higher creatinine in the HFsnEF group. However, after calculating the creatinine clearance based on age and weight, there was no significant difference in renal function between the groups. Patients in the HFmrEF group were more likely to be chronically treated with guideline directed medical therapy for HFrEF (beta-blockers, angiotensin-converting enzyme inhibitors, SGLT2i, angiotensin receptor-neprilysin inhibitors) as well as with antiplatelets. The use of loop diuretics, as well as furosemide daily doses, were similar in all 3 groups.

**Table 1:** Baseline characteristics by LVEF category

	HFmrEF (n = 750)		HFpEF (n = 4360)		HFsnEF (n = 1092)		Total (n = 6202)		P value	
Demographics and clinical data										
Age	77.8 ± 16.3		81.3 ± 13.8		84.2 ± 12.7		81.4 ± 14.1		< 0.001^a^	
Female sex	267 (35.6%)		2245 (51.5%)		747 (68.4%)		3259 (52.5%)		< 0.001^b^	
BMI – k/m^2^	28.3 ± 6.2		30.2 ± 6.9		29.9 ± 6.2		29.9 ± 6.7		< 0.001^a^	
IHD	379 (50.5%)		1461 (33.5%)		301 (27.6%)		2141 (34.5%)		< 0.001^b^	
Diabetes	302 (40.3%)		1650 (37.8%)		400 (36.6%)		2352 (37.9%)		0.281^b^	
Hypertension	463 (61.7%)		2826 (64.8%)		788 (72.2%)		4077 (65.7%)		< 0.001^b^	
Dyslipidemia	193 (25.7%)		1085 (24.9%)		286 (26.2%)		1564 (25.2%)		0.653^b^	
CKD	149 (19.9%)		744 (17.1%)		179 (16.4%)		1072 (17.3%)		0.119^b^	
Stroke	156 (20.8%)		792 (18.2%)		199 (18.2%)		1147 (18.5%)		0.222^b^	
Atrial fibrillation	290 (38.7%)		1858 (42.6%)		433 (39.7%)		2581 (41.6%)		0.045^b^	
Dementia	14 (1.9%)		117 (2.7%)		43 (3.9%)		174 (2.8%)		0.020^b^	
COPD	84 (11.2%)		608 (13.9%)		172 (15.8%)		864 (13.9%)		0.021^b^	
Anemia	168 (22.4%)		965 (22.1%)		248 (22.7%)		1381 (22.3%)		0.915^b^	
Malignancy	107 (14.3%)		652 (15.0%)		161 (14.7%)		920 (14.8%)		0.883^b^	
Smoking	103 (13.7%)		525 (12.0%)		122 (11.2%)		750 (12.1%)		0.249^b^	
SBP – mmHg	136.3 ± 26.1		140.1 ± 26.8		142.8 ± 27.2		140.1 ± 26.8		< 0.001^a^	
Heart rate – bpm	80.8 ± 19.5		79.2 ± 18.5		79.7 ± 18.0		79.5 ± 18.5		0.109^a^	
Laboratory results										
Hemoglobin—g/dL	11.7 ± 2		11.5 ± 2		11.4 ± 2		11.5 ± 2		0.030^a^	
Creatinine—mg/dL	1.2 [0.9, 1.7]		1.1 [0.9, 1.6]		1.1 [0.8, 1.5]		1.1 [0.9, 1.6]		< 0.001^c^	
Creatinine clearance—mL/min^e^	57.2 ± 26.5		56.2 ± 24.9		57.1 ± 24.5		56.5 ± 25.1		0.508^a^	
Albumin—g/dL	3.6 ± 0.5		3.6 ± 0.5		3.6 ± 0.5		3.6 ± 0.5		0.989^a^	
TSH – mIU/L	2.0 [1.2, 3.2]		2.1 [1.3, 3.5]		2.2 [1.3, 3.6]		2.1 [1.3, 3.5]		0.043^c^	
T4 – pmol/L	12.7 [11.1, 15.1]		12.9 [11.1, 15.1]		13.0 [11.2, 15.1]		12.9 [11.1, 15.1]		0.514^c^	
T3 – pmol/L	4.2 ± 1.2		4.1 ± 1		4.0 ± 0.8		4.1 ± 1		0.051^a^	
Chronic medications										
Beta-blockers	583 (77.7%)		3207 (73.6%)		762 (69.8%)		4552 (73.4%)		0.001^b^	
ACEi	540 (72.0%)		2930 (67.2%)		738 (67.6%)		4208 (67.8%)		0.033^b^	
MRA	192 (25.6%)		1235 (28.3%)		271 (24.8%)		1698 (27.4%)		0.034^b^	
ARNI	11 (1.5%)		20 (0.5%)		0 (0.0%)		31 (0.5%)		< 0.001^b^	
SGLT2i	23 (3.1%)		99 (2.3%)		14 (1.3%)		136 (2.2%)		0.030^b^	
Furosemide	516 (68.8%)		3177 (72.9%)		797 (73.0%)		4490 (72.4%)		0.063^b^	
Furosemide dose—mg	40.0 [40.0, 40.0]		40.0 [40.0, 40.0]		40.0 [40.0, 40.0]		40.0 [40.0, 40.0]		0.259^c^	
Thiazide	128 (17.1%)		952 (21.8%)		246 (22.5%)		1326 (21.4%)		0.008^b^	
Antiarrhythmics	128 (17.1%)		794 (18.2%)		203 (18.6%)		1125 (18.1%)		0.689^b^	
Digoxin	53 (7.1%)		313 (7.2%)		67 (6.1%)		433 (7.0%)		0.479^b^	
Anti platelet	492 (65.6%)		2474 (56.7%)		615 (56.3%)		3581 (57.7%)		< 0.001^b^	
Anticoagulation	340 (45.3%)		2238 (51.3%)		517 (47.3%)		3095 (49.9%)		0.002^b^	
Metformin	198 (26.4%)		1172 (26.9%)		277 (25.4%)		1647 (26.6%)		0.595^b^	
Statins	492 (65.6%)		2818 (64.6%)		704 (64.5%)		4014 (64.7%)		0.861^b^	
Echo parameters										
LVEF—%	0 (0%)	45.0 ± 1.3		57.1 ± 3.9		66.5 ± 2.8		57.3 ± 6.7		< 0.001^a^
Left ventricle end-diastolic diameter – cm	40 (0.6%)	5.1 ± 0.6		4.7 ± 0.6		4.5 ± 0.6		4.7 ± 0.6		< 0.001^a^
Interventricular septum thickness – cm	48 (0.8%)	1.1 ± 0.3		1.2 ± 0.3		1.2 ± 0.3		1.2 ± 0.3		< 0.001^a^
Left ventricle posterior wall thickness – cm	54 (0.9%)	1.1 ± 0.6		1.1 ± 0.6		1.1 ± 0.5		1.1 ± 0.6		0.340^a^
Left atrium volume index—ml/m^2^	4186 (67.5%)	42.5 ± 20.2		46.2 ± 31.9		46.5 ± 21.8		45.8 ± 29.3		0.197^a^
Tissue doppler e/e’ ratio lateral	236 (4%)	13.2 ± 7.8		14.2 ± 7.7		15.9 ± 8.1		14.4 ± 7.8		< 0.001^a^
Tissue doppler e/e’ ratio septal	1353 (21.8%)	18.2 ± 9.4		18.7 ± 9.6		20.1 ± 10.4		18.9 ± 9.7		< 0.001^a^
Mitral regurgitation – moderate to severe	662 (10.7%)	129 (18.9%)		582 (14.9%)		156 (16.6%)		867 (15.6%)		0.020^b^
Systolic pulmonary artery pressure—mmHg	855 (13.8%)	44.4 ± 15.1		47.6 ± 15.5		49.7 ± 17.1		47.6 ± 15.8		< 0.001^a^
Dilated right ventricle	3652 (58.9%)	70 (22.8%)		538 (28.1%)		93 (28.5%)		701 (27.5%)		0.144^b^
Right ventricular dysfunction – moderate to severe	3676 (59.3%)	51 (16.7%)		309 (16.3%)		51 (15.6%)		411 (16.3%)		0.929^b^
Tricuspid regurgitation – moderate to severe	402 (6.5%)	131 (18.8%)		930 (22.7%)		207 (20.5%)		1268 (21.9%)		0.036^b^

^a^One-way ANOVA,

^b^Chi-square test,

^c^Kruskal-Wallis test,

^d^Linear-by-Linear association,

^e^calculated by CKD-EPI formula

*HFmrEF* heart failure with mildly reduced ejection fraction, *HFpEF* heart failure with preserved ejection fraction, *HFsnEF* heart failure with supranormal ejection fraction, *IHD* ischemic heart disease, *BMI* body mass index, *CKD* chronic kidney disease, *COPD* chronic obstructive pulmonary disease, *SBP* systolic blood pressure, *CRT* cardiac resynchronization therapy, *TSH* thyroid stimulating hormone, *GFR* glomerular filtration rate, *SGLT*2*i* sodium-glucose co-transporter 2 Inhibitors, *ACEi* angiotensin-converting enzyme inhibitors, *ARNI* Angiotensin receptor-neprilysin inhibitors, *LVEF* left ventricular ejection fraction, *MRA* mineralocorticoid receptor antagosits

The mean LVEF was 45.0 ± 1.3 in the HFmrEF group, 57.1 ± 3.9 in the HFpEF group, and 66.5 ± 2.8 in the HFsnEF group (p < 0.001). The HFsnEF group exhibited a significantly smaller LV end diastolic diameter (LVEDD), elevated E/e’ ratio, and higher systolic pulmonary artery pressure (SPAP), compared to the other groups (p < 0.001 for all) (Table 1).

We also performed a sub-analysis to compare all 3 HF groups in the inpatient and outpatient cohorts, as well a dedicated comparison of the HFsnEF inpatients and outpatients. The results are presented in supplementary tables S1-S3**.** HFsnEF patients were older, with female predominance and a higher prevalence of hypertension as well as more severe LV hypertrophy, smaller LVEDD, elevated E/e’ ratio and higher SPAP compared to each of the other groups in both inpatient and outpatient cohorts (p < 0.001 for all). Moreover, hospitalized HFsnEF patients were older and had more comorbidities. While no difference was observed in the EF between hospitalized and ambulatory HFsnEF patients, the LV dimensions were smaller, and the lateral e/e’ ratio and SPAP were higher in the inpatient HFsnEF group.

In the inpatient cohort, there were no significant differences between all 3 HF groups regarding the possible confounders for hyperdynamic LV that were examined, including anemia (p = 0.088) and sepsis (p = 0.137). Among this cohort, the rates of echocardiographic studies conducted during hospitalization were slightly higher in the HFmrEF group (73% compared to 67.1% in the HFpEF and 68.3% in the HFsnEF groups, p = 0.035) (Table S4).

### Outcomes

**Fig. 1:**
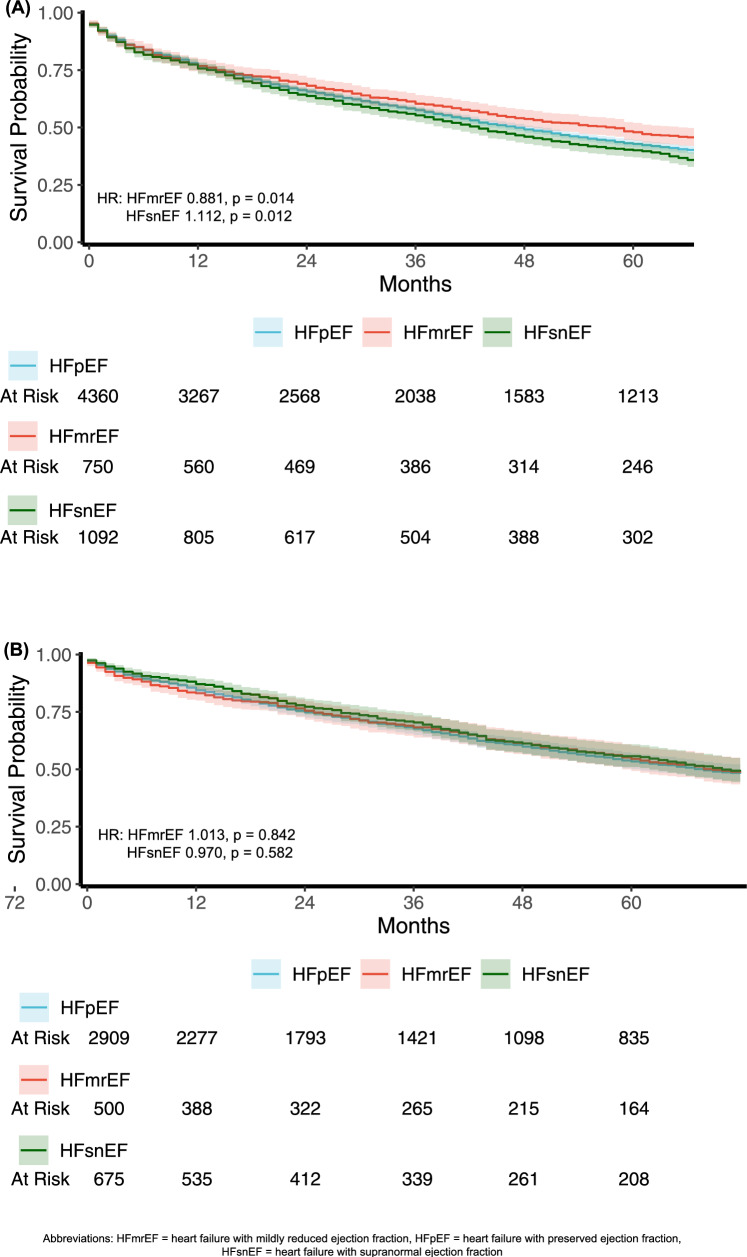
Survival plots: univariable vs. multivariable COX models: Time to death by LVEF category. **A** survival plot of time to death by LVEF category, **B** survival plot of time to death by LVEF, adjusted to age, sex, BMI, IHD, HTN, Dementia, COPD, MR and TR

The total frequency of all-cause mortality in our cohort was 3802 (61.3%), with 439 (58.5%) in the HFmrEF group, 2652 (60.8%) in the HFpEF group, and 3802 (61.3%) in the HFsnEF group. The difference between the groups was statistically significant (p < 0.001). During a median follow-up period of 32 (IQR 11–65) months, the mortality rate in the HFsnEF group was significantly higher compared to the other groups: 711 (65.1%) vs. 2,652 (60.8%) in the HFpEF group and 439 (58.5%) in the HFmrEF group (p = 0.009). A univariable Kaplan–Meier table with the Log-rank test supported this finding, with lower mean and median survival time in the HFsnEF group (p = 0.001) (Fig. 1A). In a univariable COX regression model, the mortality rates were higher in the HFsnEF group compared to HFmrEF and HFpEF patients (HR 1.258, 95% CI 1.117–1.418; p < 0.001 and HR 1.112, 95% CI 1.023–1.208; p = 0.012, respectively) (Table 2). However, after adjusting for baseline characteristics, comorbidities, and echocardiographic parameters, there was no significant difference in the mortality rates between the groups (Table 2 and Fig. 1B).

**Table 2:** Univariable and multivariable COX regression models: Time to death by various independent variables

	Unadjusted HR (95% CI)	95%CI	P-value	Adjusted HR (95% CI)	95%CI	P value
LVEF category						
HFpEF (reference)			0.001			0.826
HFmrEF	0.881	0.797–0.975	0.014	1.014	0.891–1.153	0.862
HFsnEF	1.258	1.117–1.418	0.012	0.963	0.871–1.081	0.581
Age	1.047	1.044–1.050	< 0.001	1.04	1.040–1.048	< 0.001
Female sex	1.113	1.044–1.186	0.001	0.901	0.857–1.013	0.05
BMI	0.988	0.983–0.944	< 0.001	0.992	0.988–1.002	0.082
Sepsis	1.830	1.703–1.967	< 0.001			
IHD	1.243	1.164–1.327	< 0.001	1.089	0.999–1.186	0.107
Hypertension	1.353	1.261–1.452	< 0.001	1.040	0.948–1.141	0.781
CKD	1.738	1.609–1.877	< 0.001			
Atrial fibrillation	1.303	1.223–1.389	< 0.001			
Dementia	2.134	1.811–2.514	< 0.001	1.483	1.177–1.869	0.039
COPD	1.460	1.342–1.588	< 0.001	1.437	1.290–1.601	< 0.001
Hemoglobin – g/dL	0.883	0.869–0.898	< 0.001			
Creatinine – mg/dL	1.138	1.110–1.167	< 0.001			
SPAP – mmHg	1.021	1.019–1.023	< 0.001			
Mitral regurgitation	1.252	1.145–1.369	< 0.001	0.968	0.867–1.081	0.05
Tricuspid regurgitation	1.832	1.701–1.972	< 0.001	1.573	1.430–1.729	< 0.001

In bold: variables that were included in the multivariable analysis. *BMI* body mass index, *IHD* ischemic heart disease, *CKD* chronic kidney disease, *COPD* chronic obstructive pulmonary disease, *SPAP* systolic pulmonary artery pressure; *TSH* thyroid stimulating hormone

During the entire follow-up period, there were 4,100 readmissions for all-cause hospitalization, with 2922 (67.0%) in the HFpEF group, 455 (60.7%) in the HFmrEF group, and 723 (66.2%) in the HFsnEF group. The difference between the groups was statistically significant (χ^2^ = 11.529, p = 0.003). A Kaplan–Meier table with the Log-rank test revealed lower total hospitalization rates in the HFmrEF group compared with the HFpEF and HFsnEF groups, as manifested by an increased median duration of freedom from total hospitalizations (1.7 [0.99, 2.4] months vs 1.1 [0.9, 1.2] months and 1.1 [0.8, 1.4] months, respectively; p = 0.022).

**Fig. 2:**
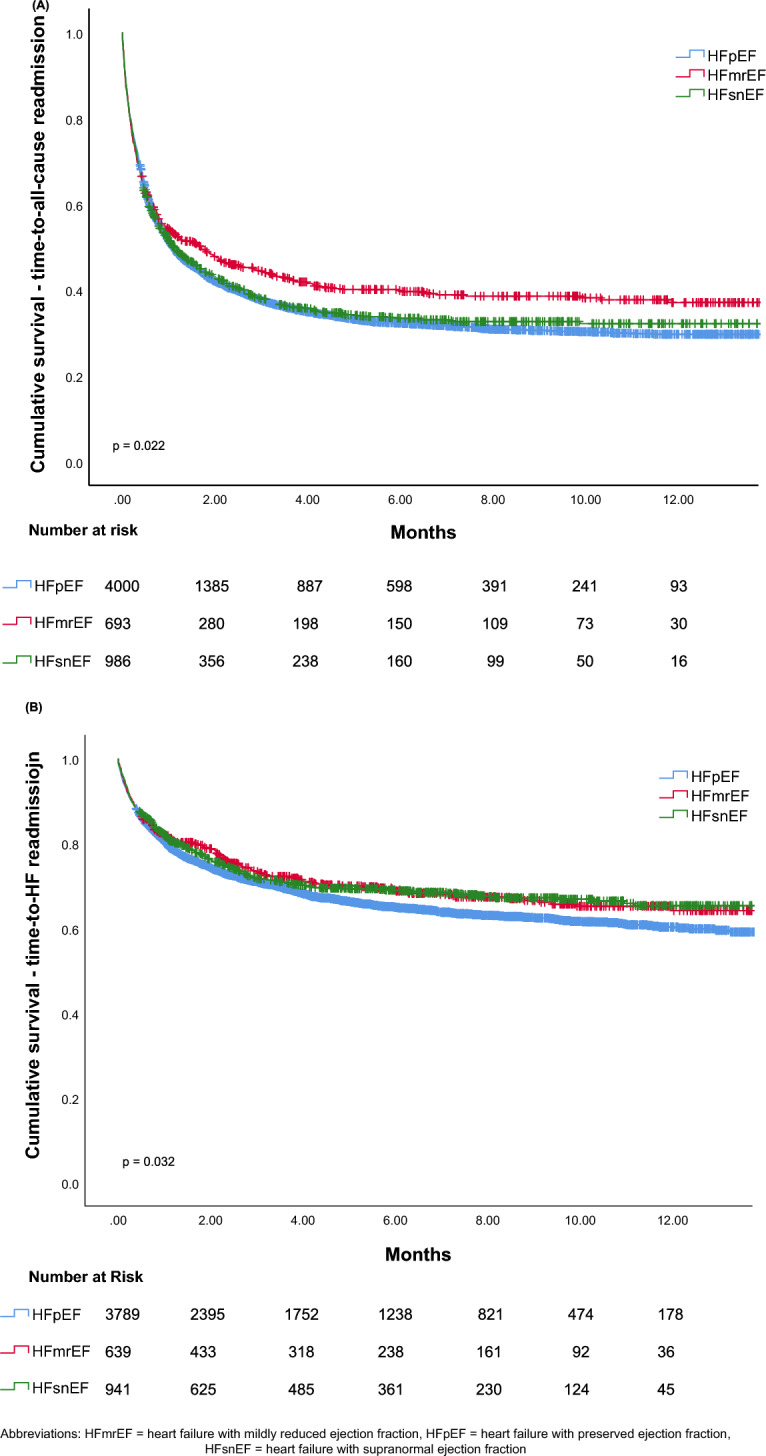
Time to hospitalization by LVEF category. **A** survival plot of time to all-cause hospitalization by LVEF category, **B** survival plot of time to heart failure related hospitalization by LVEF category

As for HF readmissions, the total number was 2,419 (39.0%), with 1,737 (39.8%) in the HFpEF group, 282 (37.6%) in the HFmrEF group, and 400 (36.6%) in the HFsnEF group. The difference between the groups was not statistically significant (χ^2^ = 4.487, p = 0.106). A Kaplan–Meier table with the Log-rank test revealed higher HF-related hospitalization rates in the HFpEF group, as evidenced by a lower average duration of freedom from HF-related hospitalizations (9.6 [9.4–9.9] months vs 10.1 [9.6–10.6]months and 10.2 [9.8–10.6], in the HFmrEF and HFsnEF groups respectively; p = 0.0032, Fig. 2).

## Discussion

Since its conceptualization, this is the largest study to evaluate the clinical characteristics and outcomes of patients with HFsnEF compared to patients with HFpEF and HFmrEF in a contemporary cohort including both hospitalized and ambulatory HF patients. Our results demonstrate a distinct clinical profile of HFsnEF patients compared to the other groups as well as more pronounced cardiac structural and diastolic abnormalities. Moreover, we found increased mortality rates within patients with HFsnEF, compared to patients with HFpEF and HFmrEF. However, in a multivariable model, adjusted for age, sex, and comorbidities, there was no significant difference in all-cause mortality rates between all groups.

Our data demonstrates differences in the baseline characteristics between study groups. Patients with HFsnEF were significantly older, more likely to be females and had higher prevalence of hypertension, compared to patients with HFpEF and HFmrEF. These findings are in line with previous reports [11, 13, 14] and reflect fundamental characteristics of this group. Moreover, we witnessed higher rates of COPD in the HFsnEF group, a common comorbidity in HFpEF patients [15, 16]. Despite lack of robust data, the associated LV hypertrophy in patients with COPD could theoretically explain the numerically higher LVEF observed [17]. In older subjects, a higher LVEF may result from an increased arterial stiffening, greater LV mass, and reduced LV dimensions, which require higher global contractility [18–20]. In women, it can act as a compensatory mechanism for smaller indexed LV volumes [11, 13, 14, 21–23], potentially accounting for the gender distribution observed in our study across the different HF subgroups. And indeed, as low ventricular volumes with preserved stroke volume mathematically result in an increased EF, females have a higher threshold for normal EF [24]. This potentially limits EF measurement and its ability to differentiate a pathological process from a physiological one. However, it has been shown that, outside the context of HF, women with snEF have more microvascular dysfunction, increased sympathetic tone, reduced pumping efficiency and a higher risk of major adverse cardiac events and mortality [13, 25, 26]. These data suggest an underlying pathological process that may contribute to worse outcomes. Therefore, we addressed the female sex specifically in our multivariable model. Although female sex was associated with increased all-cause mortality in the univariable analysis, it was not in the multivariable model, suggesting a secondary effect via other components. Further investigation of this interesting subgroup of women with HF and snEF may be of great interest. The consistency of higher hypertension prevalence [14, 23], alongside a greater extent of LV hypertrophy, suggest a strong phenotypical and possibly mechanistic relationship between hypertension and HFsnEF.

Conversely, the HFmrEF group exhibited higher rates of IHD and accordingly higher likelihood of receiving chronic antiplatelet therapy, which suggests a similarity to HFrEF populations, characterized by an increased prevalence of ischemic cardiomyopathy [27]. Despite differences in the pathophysiology between HF with reduced vs. preserved EF, some of the above-mentioned clinical and echocardiographic parameters, both in our as well as in previous large-scale studies [9, 11], presented a graded effect, such that they progressively increased with the transition from HFmrEF to HFpEF and HFsnEF. Therefore, our findings may imply an already known similarity between HFmrEF and HFrEF patients, but also between HFsnEF and HFpEF, with the former potentially representing an extreme manifestation of HFpEF characteristics. Further research is needed to explore the potential pathophysiology of HFsnEF and its possible relationship to HFpEF.

The echocardiographic evaluation of HF patients may differ in the acute versus the chronic setting [21]. One of the unique features of our cohort is the inclusion of ambulatory stable patients (n = 908 [15%]). Hence, we performed several sub-analyses to address this issue. A separate analysis of the inpatients and outpatients cohorts demonstrated similar trends in baseline characteristics, laboratory parameters, chronic medications, and echocardiographic variables. This important observation suggests that similar clinical parameters characterize both hospitalized as well as chronic ambulatory HFsnEF patients and is in line with previous reports [9]. To mitigate potential confounders that might enhance the LVEF during hospitalization, we examined the incidence of severe anemia, sepsis and acute bleeding among the inpatient cohort. However, all these factors were present at comparable rates among the different study groups. Moreover, the rate of echocardiographic studies performed during hospitalization was similar between the HFpEF and HFsnEF groups. These findings support the notion that the increased LVEF within the hospitalized cohort is less likely to be a result of an acute transient events and, therefore, more accurately represents the HFsnEF phenotype.

In the current study, patients with HFsnEF had higher rates of all-cause mortality. Nevertheless, an adjusted multivariable model revoked the notion that snEF is an independent risk factor for mortality, implying an indirect effect on the outcome through other parameters. The association between snEF and mortality remains controversial, as prior studies demonstrated conflicting results. Some of the studies that suggested higher mortality with snEF have several important limitations. Crucially, most of these trials did not include HF patients and therefore address the issue of snEF in the general population [10, 14, 23]. Two previous studies evaluated outcomes in patients with HFsnEF. The first was a subanalysis of the RELAX-AHF-2 trial, including 6128 acute HF patients, which showed that acute HFsnEF patients exhibited comparable rates of both all-cause mortality and CV death, compared to both HFpEF and HFmrEF patients, with a higher risk of non-cv death. Importantly, all patients were hospitalized with an acute HF event and the HFsnEF population in this study cohort constituted a small minority and included only 155 (2.5%) patients, limiting the generalizability of these findings [11]. Greater proportion of non-CV death among patients with higher LVEF (≥ 60%) was also reported in a different study of chronic HF patients [7]. These findings are consistent with our study results and allude to non-cardiac baseline comorbidities as a potential cause that limit the survival of these patients. The largest study thus far to also include HF patients, by Wehner et al., suggested a U-shaped relationship between LVEF and mortality with significantly higher adjusted HR for both inpatients and outpatients in the HFsnEF group [9]. However, the vast majority of patients in this study (87%) did not have a diagnosis of HF and therefore these findings should be viewed as exploratory. In addition, the adjusted models did not address important clinical factors that were included in our model such as IHD, hypertension, BMI and dementia. Moreover, the significant association between HFsnEF and all-cause mortality was blunted when further adjusted for NT-proBNP levels, suggesting that increased mortality stems from the severity of the HF disease rather than from the LVEF itself. Our findings suggests that HFsnEF itself is not an independent predictor of worse outcomes, but rather, the higher mortality is driven by the older age and comorbidity burden of this group.

In our study, patients with HFmrEF exhibited lower rates of total hospitalizations compared to the other groups, consistent with previous reports that have compared HFmrEF with HFpEF (24). Importantly, although there was no significant difference in the rates of all-cause hospitalizations between the HFpEF and HFsnEF groups, the HFsnEF group exhibited lower rates of HF-related hospitalizations, suggesting a higher proportion of non-HF hospitalizations in this group. This further supports the notion that the observed poor prognosis in the HFsnEF group may be attributed to non-HF comorbidities.

### Limitations

We acknowledge several limitations to our study. First, this is an observational study with a retrospective analysis of collected data. Secondly, the study’s generalizability may be restricted since it was carried out in a single tertiary medical center, potentially introducing patient selection bias. In addition, data on natriuretic peptides levels were missing in a substantial portion of our cohort and therefore was not included in the multivariable analysis model. Lastly, hospitalizations were considered in our center alone. However, due to the high hospitalization rate (over 60% in the entire cohort), we believe our findings represent the actual hospitalization rate.

## Conclusions

Our study demonstrates that HFsnEF patients exhibit a distinct clinical and echocardiographic profile compared to patients with HFmrEF and HFpEF. Moreover, despite increased all-cause mortality within HFsnEF patients in a univariable analysis, adjusted mortality risk was similar among all 3 HF phenotypes, suggesting that the lower survival is not directly mediated by the higher LVEF. Therefore, patients with HFsnEF should be perceived as having a higher risk for worse outcome compared to HFmrEF and HFpEF patients. Nevertheless, HFsnEF may serve as an indicator for underlying comorbidities rather than an independent prognostic factor.

## Supplementary Information

Below is the link to the electronic supplementary material.

Supplementary file1 (DOCX 48 KB)

## Data Availability

The datasets generated during and analysed during the current study are available from the corresponding author on reasonable request.
