# Supplementary material for: Heart failure with supranormal ejection fraction: clinical characteristics and outcomes compared to mildly reduced and preserved ejection fraction
Source: Clin Res Cardiol. 2025 Feb 24;114(5):665–75. doi: 10.1007/s00392-025-02620-9 (PMC12058924; doi:10.1007/s00392-025-02620-9)
Supplement: Supplementary file 1 — Supplementary file1 (DOCX 48 KB) [file 392_2025_2620_MOESM1_ESM.docx]

# Table S1 – Baseline characteristics of inpatients by LVEF group

| Demographics and clinical data | | | | | |
| --- | --- | --- | --- | --- | --- |
|  | **HFmrEF  (n = 589)** | **HFpEF (n = 3749)** | **HFsnEF (n = 956)** | **Total (n = 5294)** | **P-value** |
| Age | 81.1 ± 13.9 | 83.0 ± 12.4 | 85.7 ± 11.1 | 83.3 ± 12.4 | <0.001^a^ |
| Female sex | 206 (35.0%) | 1956 (52.2%) | 665 (69.6%) | 2827 (53.4%) | <0.001^b^ |
| BMI | 28.5 ± 6.3 | 30.5 ± 7 | 29.8 ± 6.4 | 30.1 ± 6.9 | <0.001^a^ |
| IHD | 341 (57.9%) | 1328 (35.4%) | 279 (29.2%) | 1948 (36.8%) | <0.001^b^ |
| Diabetes | 261 (44.3%) | 1483 (39.6%) | 359 (37.6%) | 2103 (39.7%) | 0.029^b^ |
| Hypertension | 400 (67.9%) | 2505 (66.8%) | 700 (73.2%) | 3605 (68.1%) | 0.001^b^ |
| Dyslipidemia | 173 (29.4%) | 965 (25.7%) | 260 (27.2%) | 1398 (26.4%) | 0.147^b^ |
| CKD | 124 (21.1%) | 671 (17.9%) | 161 (16.8%) | 956 (18.1%) | 0.101^b^ |
| Stroke | 139 (23.6%) | 729 (19.4%) | 187 (19.6%) | 1055 (19.9%) | 0.061^b^ |
| Atrial fibrillation | 247 (41.9%) | 1646 (43.9%) | 381 (39.9%) | 2274 (43.0%) | 0.068^b^ |
| Dementia | 14 (2.4%) | 116 (3.1%) | 40 (4.2%) | 170 (3.2%) | 0.111^b^ |
| COPD | 79 (13.4%) | 563 (15.0%) | 159 (16.6%) | 801 (15.1%) | 0.216^b^ |
| Anemia | 146 (24.8%) | 855 (22.8%) | 225 (23.5%) | 1226 (23.2%) | 0.544^b^ |
| Malignancy | 81 (13.8%) | 554 (14.8%) | 142 (14.9%) | 777 (14.7%) | 0.796^b^ |
| Smoking | 89 (15.1%) | 437 (11.7%) | 105 (11.0%) | 631 (11.9%) | 0.034^b^ |
| SBP (mmHg) | 139.7 ± 26.1 | 142.0 ± 27.2 | 144.0 ± 27.8 | 142.1 ± 27.2 | 0.009^a^ |
| Heart rate (bpm) | 82.5 ± 19.9 | 80.0 ± 18.8 | 80.7 ± 18.2 | 80.4 ± 18.8 | 0.011^a^ |
| Max fever (°C) | 37.3 ± 0.6 | 37.2 ± 0.6 | 37.2 ± 0.6 | 37.2 ± 0.6 | 0.156^a^ |
| Labs | | | | | |
|  | **HFmrEF  (n = 589)** | **HFpEF (n = 3749)** | **HFsnEF (n = 956)** | **Total (n = 5294)** | **P-value** |
| Hemoglobin (g/dL) | 11.6 ± 2.0 | 11.4 ± 1.9 | 11.3 ± 1.9 | 11.4 ± 2.0 | 0.069^a^ |
| Creatinine (mg/dL) | 1.2 [0.9, 1.6] | 1.1 [0.9, 1.6] | 1.1 [0.8, 1.5] | 1.1 [0.9, 1.6] | <0.001^c^ |
| Creatinine clearance (mL/min^e^) | 56.6 ± 26 | 55.6 ± 24.5 | 56.6 ± 24.2 | 55.9 ± 24.6 | 0.441^a^ |
| Albumin – g/dL | 3.6 ± 0.5 | 3.6 ± 0.4 | 3.6 ± 0.5 | 3.6 ± 0.5 | 0.938^a^ |
| TSH – mIU/L | 1.9 [1.2, 3.2] | 2.1 [1.3, 3.5] | 2.2 [1.3, 3.6] | 2.1 [1.3, 3.5] | 0.022^c^ |
| T4 – pmol/L | 12.7  [11.2, 15.4] | 13.0  [11.2, 15.1] | 13.0  [11.2, 15.2] | 12.9 [11.2, 15.2] | 0.653^c^ |
| T3 – pmol/L | 4.1 ± 1.2 | 4.0 ± 1.0 | 4.0 ± 0.8 | 4.0 ± 1.0 | 0.095^a^ |
| Chronic medications | | | | | |
|  | **HFmrEF  (n = 589)** | **HFpEF (n = 3749)** | **HFsnEF (n = 956)** | **Total (n = 5294)** | **P-value** |
| Beta-blockers | 449 (76.2%) | 2782 (74.2%) | 670 (70.1%) | 3901 (73.7%) | 0.012^b^ |
| ACEi | 422 (71.6%) | 2550 (68.0%) | 653 (68.3%) | 3625 (68.5%) | 0.210^b^ |
| Aldosterone | 142 (24.1%) | 999 (26.6%) | 225 (23.5%) | 1366 (25.8%) | 0.089^b^ |
| ARNI | 3 (0.5%) | 6 (0.2%) | 0 (0.0%) | 9 (0.2%) | 0.024^b^ |
| SGLT2i | 20 (3.4%) | 92 (2.5%) | 13 (1.4%) | 123 (2.4%) | 0.030^b^ |
| Furosemide | 432 (73.3%) | 2836 (75.6%) | 730 (76.4%) | 3998 (75.5%) | 0.386^b^ |
| Furosemide dose   (mg) | 40.0  [40.0, 40.0] | 40.0  [40.0, 40.0] | 40.0  [40.0, 40.0] | 40.0  [40.0, 40.0] | 0.158^c^ |
| Thiazide | 113 (19.2%) | 843 (22.5%) | 210 (22.0%) | 1166 (22.0%) | 0.199^b^ |
| Antiarrhythmics | 99 (16.8%) | 698 (18.6%) | 181 (18.9%) | 978 (18.5%) | 0.530^b^ |
| Digoxin | 37 (6.3%) | 266 (7.1%) | 59 (6.2%) | 362 (6.8%) | 0.511^b^ |
| Anti platelet | 427 (72.5%) | 2196 (58.6%) | 559 (58.5%) | 3182 (60.1%) | <0.001^b^ |
| Anticoagulation | 291 (49.4%) | 1993 (53.2%) | 459 (48.0%) | 2743 (51.8%) | 0.008^b^ |
| Metformin | 170 (28.9%) | 1059 (28.2%) | 250 (26.2%) | 1479 (27.9%) | 0.378^b^ |
| Statins | 422 (71.6%) | 2496 (66.6%) | 628 (65.7%) | 3546 (67.0%) | 0.033^b^ |
| Echo parameters | | | | | |
|  | **HFmrEF  (n = 589)** | **HFpEF (n = 3749)** | **HFsnEF (n = 956)** | **Total (n = 5294)** | **P-value** |
| LVEF - % | 45.0 ± 1.3 | 57.1 ± 3.8 | 66.4 ± 2.7 | 57.5 ± 6.6 | <0.001^a^ |
| Left ventricle end-diastolic diameter – cm | 5.1 ± 0.6 | 4.7 ± 1.4 | 4.5 ± 1.5 | 4.7 ± 1.4 | <0.001^a^ |
| Interventricular septum thickness – cm | 1.2 ± 0.4 | 1.2 ± 0.3 | 1.2 ± 0.2 | 1.2 ± 0.3 | <0.001^a^ |
| Left ventricle posterior wall thickness – cm | 1.1 ± 0.7 | 1.1 ± 0.6 | 1.1 ± 0.4 | 1.1 ± 0.6 | 0.521^a^ |
| Left atrium volume index -ml/m² | 44.9 ± 21.4 | 47.5 ± 33.7 | 46.9 ± 22.5 | 47.1 ± 31 | 0.586^a^ |
| Tissue doppler E/e’ ratio lateral | 14.2 ± 8.2 | 14.5 ± 7.8 | 16.1 ± 8.3 | 14.8 ± 8 | <0.001^a^ |
| Tissue doppler E/e’ ratio septal | 19.5 ± 9.7 | 19 ± 9.3 | 20.4 ± 10.6 | 19.3 ± 9.6 | 0.003^a^ |
| Mitral regurgitation –  moderate to severe | 114 (20.5%) | 529 (15.5%) | 138 (16.5%) | 781 (16.2%) | 0.012^b^ |
| Systolic pulmonary artery pressure – mmHg | 45.9 ± 15.1 | 48.5 ± 15.3 | 50.5 ± 17.1 | 48.6 ± 15.7 | <0.001^a^ |
| Dilated right ventricle | 60 (24.4%) | 497 (29.6%) | 87 (29.7%) | 644 (29.0%) | 0.235^b^ |
| Right ventricular dysfuncation – moderate to severe | 45 (18.1%) | 287 (17.3%) | 48 )16.4%) | 380 (17.3%) | 0.861^b^ |
| Tricuspid regurgitation – moderate to severe | 112 (20.0%) | 857 (24.1%) | 190 (21.3%) | 1159 (23.1%) | 0.037^b^ |

^a^- One-way ANOVA, ^b^- Chi-square test, ^c^- Kruskal-Wallis test, ^d^-Linear-by-Linear association, ^e^-calculated by CKD-EPI formula

Abbreviations: HFmrEF = heart failure with mildly reduced ejection fractions, HFpEF = heart failure with preserved ejection fraction, HFsnEF = heart failure with supranormal ejection fraction, IHD = ischemic heart disease, CKD = chronic kidney disease, COPD = chronic obstructive pulmonary disease, SBP = systolic blood pressure, TSH = thyroid stimulating hormone, GFR = glomerular filtration rate, SGLT2i = sodium-glucose co-transporter 2 Inhibitors, ACEi = angiotensin-converting enzyme inhibitors, ARNI = Angiotensin receptor-neprilysin inhibitors, LVEF = left ventriculat ejection fraction.

# Table S2 – Baseline characteristics of outpatients by LVEF group

| Demographics and clinical data | | | | | |
| --- | --- | --- | --- | --- | --- |
|  | **HFmrEF  (n = 161)** | **HFpEF  (n = 611)** | **HFsnEF**  **(n = 136)** | **Total**  **(n = 908)** | **P-value** |
| Age | 65.9 ± 18.5 | 70.9 ± 17 | 73.5 ± 17.2 | 70.4 ± 17.5 | <0.001^a^ |
| Female sex | 61 (37.9%) | 289 (47.3%) | 82 (60.3%) | 432 (47.6%) | 0.001^b^ |
| BMI | 27.8 ± 5.9 | 28.9 ± 5.8 | 30.0 ± 5.4 | 28.9 ± 5.8 | 0.006^a^ |
| IHD | 38 (23.6%) | 133 (21.8%) | 22 (16.2%) | 193 (21.3%) | 0.256^b^ |
| Diabetes | 41 (25.5%) | 167 (27.3%) | 41 (30.1%) | 249 (27.4%) | 0.664^b^ |
| Hypertension | 63 (39.1%) | 321 (52.5%) | 88 (64.7%) | 472 (52.0%) | <0.001^b^ |
| Dyslipidemia | 20 (12.4%) | 120 (19.6%) | 26 (19.1%) | 166 (18.3%) | 0.104^b^ |
| CKD | 25 (15.5%) | 73 (11.9%) | 18 (13.2%) | 116 (12.8%) | 0.473^b^ |
| Stroke | 17 (10.6%) | 63 (10.3%) | 12 (8.8%) | 92 (10.1%) | 0.857^b^ |
| Atrial fibrillation | 43 (26.7%) | 212 (34.7%) | 52 (38.2%) | 307 (33.8%) | 0.081^b^ |
| Dementia | 0 (0.0%) | 1 (0.2%) | 3 (2.2%) | 4 (0.4%) | 0.006^d^ |
| COPD | 5 (3.1%) | 45 (7.4%) | 13 (9.6%) | 63 (6.9%) | 0.071^b^ |
| Anemia | 22 (13.7%) | 110 (18.0%) | 23 (16.9%) | 155 (17.1%) | 0.428^b^ |
| Malignancy | 26 (16.1%) | 98 (16.0%) | 19 (14.0%) | 143 (15.7%) | 0.826^b^ |
| Smoking | 14 (8.7%) | 88 (14.4%) | 17 (12.5%) | 119 (13.1%) | 0.158^b^ |
| SBP – mmHg | 124.1 ± 22.3 | 128.4 ± 20.7 | 134.1 ± 20.1 | 128.5 ± 21.1 | <0.001^a^ |
| Heart rate – bpm | 74.3 ± 16.3 | 74.6 ± 14.5 | 72.2 ± 14.7 | 74.2 ± 14.9 | 0.269^a^ |
| Labs |  |  |  |  |  |
|  | **HFmrEF  (n = 161)** | **HFpEF  (n = 611)** | **HFsnEF**  **(n = 136)** | **Total**  **(n = 908)** | **P-value** |
| Hemoglobin – g/dL | 12.0 ± 1.7 | 12.0 ± 2.0 | 12.0 ± 1.9 | 12.0 ± 2.0 | 0.991^b^ |
| Creatinine – mg/dL | 1.2 [0.9, 1.6] | 1.1 [0.9, 1.6] | 1.1 [0.8, 1.5] | 1.1 [0.9, 1.6] | <0.001^a^ |
| Creatinine clearance – mL/min^e^ | 64.5 ± 32.0 | 67.4 ± 29.4 | 68.3 ± 28.9 | 67.1 ± 29.7 | 0.833^b^ |
| Albumin – mg/dL | 3.7 ± 0.5 | 3.6 ± 0.6 | 3.7 ± 0.5 | 3.6 ± 0.5 | 0.766^b^ |
| TSH - mIU/L | 1.9 [1.2, 3.2] | 2.1 [1.3, 3.5] | 2.2 [1.3, 3.6] | 2.1 [1.3, 3.5] | 0.022^a^ |
| T4 – pmol/L | 12.7  [11.2, 15.4] | 13.0  [11.2, 15.1] | 13.0  [11.2, 15.2] | 12.9 [11.2, 15.2] | 0.653^a^ |
| T3 – pmol/L | 4.5 ± 0.9 | 4.4 ± 1 | 4.2 ± 0.8 | 4.3 ± 0.9 | 0.387^b^ |
| Chronic medications | | | | | |
|  | **HFmrEF  (n = 161)** | **HFpEF  (n = 611)** | **HFsnEF**  **(n = 136)** | **Total**  **(n = 908)** | **P-value** |
| Beta-blockers | 134 (83.2%) | 425 (69.6%) | 92 (67.6%) | 651 (71.7%) | 0.001^b^ |
| ACEi | 118 (73.3%) | 380 (62.2%) | 85 (62.5%) | 583 (64.2%) | 0.030^b^ |
| Aldosterone | 50 (31.1%) | 236 (38.6%) | 46 (33.8%) | 332 (36.6%) | 0.160^b^ |
| ARNI | 8 (5.0%) | 14 (2.3%) | 0 (0.0%) | 22 (2.4%) | 0.005^d^ |
| SGLT2i | 3 (1.9%) | 7 (1.1%) | 1 (0.7%) | 11 (1.2%) | 0.653^b^ |
| Furosemide | 84 (52.2%) | 341 (55.8%) | 67 (49.3%) | 492 (54.2%) | 0.327^b^ |
| Furosemide dose - mg | 40.0 [40.0, 40.0] | 40.0 [40.0, 40.0] | 40.0 [40.0, 40.0] | 40.0 [40.0, 40.0] | 0.749^c^ |
| Thiazide | 15 (9.3%) | 109 (17.8%) | 36 (26.5%) | 160 (17.6%) | 0.001^b^ |
| Antiarrhythmics | 29 (18.0%) | 96 (15.7%) | 22 (16.2%) | 147 (16.2%) | 0.780^b^ |
| Digoxin | 16 (9.9%) | 47 (7.7%) | 8 (5.9%) | 71 (7.8%) | 0.422^b^ |
| Anti platelet | 65 (40.4%) | 278 (45.5%) | 56 (41.2%) | 399 (43.9%) | 0.395^b^ |
| Anticoagulation | 49 (30.4%) | 245 (40.1%) | 58 (42.6%) | 352 (38.8%) | 0.049^b^ |
| Metformin | 28 (17.4%) | 113 (18.5%) | 27 (19.9%) | 168 (18.5%) | 0.862^b^ |
| Statins | 70 (43.5%) | 322 (52.7%) | 76 (55.9%) | 468 (51.5%) | 0.062^b^ |
| Echo parameters | | | | | |
|  | **HFmrEF  (n = 161)** | **HFpEF  (n = 611)** | **HFsnEF**  **(n = 136)** | **Total**  **(n = 908)** | **P-value** |
| LVEF (%) | 44.9 ± 1.3 | 56.7 ± 4.0 | 66.7 ± 2.9 | 56.1 ± 7.1 | <0.001^a^ |
| Left ventricle end-diastolic diameter – cm | 5.2 ± 0.6 | 4.9 ± 2.2 | 4.6 ± 0.5 | 4.9 ± 1.9 | 0.012^a^ |
| Interventricular septum thickness – cm | 1.1 ± 0.2 | 1.2 ± 0.4 | 1.3 ± 0.4 | 1.2 ± 0.4 | <0.001^a^ |
| Left ventricle posterior wall thickness – cm | 0.9 ± 0.2 | 1.1 ± 0.7 | 1.1 ± 0.9 | 1.1 ± 0.6 | 0.038^a^ |
| Left atrium volume index - ml/m² | 35.8 ± 14.9 | 39.8 ± 20.4 | 44.7 ± 18.3 | 39.9 ± 19.4 | 0.042^a^ |
| Tissue doppler E/e’ ratio lateral | 9.6 ± 4.7 | 12.4 ± 7 | 14.5 ± 6.3 | 12.2 ± 6.7 | <0.001^a^ |
| Tissue doppler E/e’ ratio septal | 13.4 ± 6 | 16.5 ± 10.8 | 18.6 ± 8.6 | 16.3 ± 10 | <0.001^a^ |
| Mitral regurgitation –  moderate to severe | 15 (11.8%) | 53 (10.6%) | 18 (17.1%) | 86 (11.8%) | 0.172^b^ |
| Systolic pulmonary artery pressure - mmHg | 38 ± 13.2 | 41.2 ± 15.1 | 43.9 ± 15.5 | 41.1 ± 14.9 | 0.012^a^ |
| Dilated right ventricle | 10 (16.4%) | 41 (17.2%) | 6 (18.2%) | 57 (17.2%) | 0.975^b^ |
| Right ventricular dysfuncation – moderate to severe | 6 (10.3%) | 22 (9.2%) | 3 (8.8%) | 31 (9.4%) | 0.960^b^ |
| Tricuspid regurgitation – moderate to severe | 19 (14.0%) | 73 (13.7%) | 17 (14.5%) | 109 (13.9%) | 0.974^b^ |

^a^- One-way ANOVA, ^b^- Chi-square test, ^c^- Kruskal-Wallis test, ^d^-Linear-by-Linear association, ^e^-calculated by CKD-EPI formula

Abbreviations: HFmrEF = heart failure with mildly reduced ejection fractions, HFpEF = heart failure with preserved ejection fraction, HFsnEF = heart failure with supranormal ejection fraction, IHD = ischemic heart disease, CKD = chronic kidney disease, COPD = chronic obstructive pulmonary disease, SBP = systolic blood pressure, CRT = cardiac resynchronization therapy, TSH = thyroid stimulating hormone, GFR = glomerular filtration rate, SGLT2i = sodium-glucose co-transporter 2 Inhibitors, ACEi = angiotensin-converting enzyme inhibitors, ARNI = Angiotensin receptor-neprilysin inhibitors, LVEF = left ventriculat ejection fraction.

# Table S3 – Baseline characteristics of HFsnEF patients – inpatients vs. outpatients

| **Demographics and clinical data** | | | | |
| --- | --- | --- | --- | --- |
|  | **Inpatients**  **(n = 956)** | **Outpatients**  **(n = 136)** | **Total**  **(n = 1092)** | **P-value** |
| Age | 85.7 ± 11.1 | 73.5 ± 17.2 | 84.2 ± 12.7 | <0.001^a^ |
| Female sex | 665 (69.6%) | 82 (60.3%) | 747 (68.4%) | 0.030^b^ |
| BMI | 29.8 ± 6.4 | 30.0 ± 5.4 | 29.9 ± 6.2 | 0.733^a^ |
| IHD | 279 (29.2%) | 22 (16.2%) | 301 (27.6%) | 0.001^b^ |
| Diabetes | 359 (37.6%) | 41 (30.1%) | 400 (36.6%) | 0.094^b^ |
| Hypertension | 700 (73.2%) | 88 (64.7%) | 788 (72.2%) | 0.038^b^ |
| Dyslipidemia | 260 (27.2%) | 26 (19.1%) | 286 (26.2%) | 0.045^b^ |
| CKD | 161 (16.8%) | 18 (13.2%) | 179 (16.4%) | 0.288^b^ |
| Stroke | 187 (19.6%) | 12 (8.8%) | 199 (18.2%) | 0.002^b^ |
| Atrial fibrillation | 381 (39.9%) | 52 (38.2%) | 433 (39.7%) | 0.718^b^ |
| Dementia | 40 (4.2%) | 3 (2.2%) | 43 (3.9%) | 0.267^b^ |
| COPD | 159 (16.6%) | 13 (9.6%) | 172 (15.8%) | 0.034^b^ |
| Anemia | 225 (23.5%) | 23 (16.9%) | 248 (22.7%) | 0.085^b^ |
| Malignancy | 142 (14.9%) | 19 (14.0%) | 161 (14.7%) | 0.786^b^ |
| Smoking | 105 (11.0%) | 17 (12.5%) | 122 (11.2%) | 0.599^b^ |
| SBP – mmHg | 144.0 ± 27.8 | 134.1 ± 20.1 | 142.8 ± 27.2 | <0.001^a^ |
| Heart rate – bpm | 80.7 ± 18.2 | 72.2 ± 14.7 | 79.7 ± 18.0 | <0.001^a^ |
| **Laboratory results** | | | | |
|  | **Inpatients**  **(n = 956)** | **Outpatients**  **(n = 136)** | **Total**  **(n = 1092)** | **P-value** |
| Hemoglobin - g/dL | 11.3 ± 2.0 | 11.8 ± 2.3 | 11.4 ± 2.1 | 0.040^a^ |
| Creatinine - mg/dL | 1.3 ± 0.8 | 1.2 ± 0.5 | 1.3 ± 0.8 | 0.426^a^ |
| Creatinine clearance - mL/min^e^ | 56.6 ± 24.2 | 68.3 ± 28.9 | 57.1 ± 24.5 | 0.007^a^ |
| Albumin - g/dL | 3.6 ± 0.5 | 3.7 ± 0.5 | 3.6 ± 0.5 | 0.746^a^ |
| TSH – mIU/L | 3.3 ± 4.4 | 2.9 ± 2.6 | 3.2 ± 4.3 | 0.621^a^ |
| T4 – pmol/L | 13.5 ± 3.4 | 13.2 ± 3.4 | 13.5 ± 3.4 | 0.519^a^ |
| T3 – pmol/L | 4.0 ± 0.8 | 4.2 ± 0.8 | 3.9 ± 0.8 | 0.102^a^ |
| **Chronic medications** | | | | |
|  | **Inpatients**  **(n = 956)** | **Outpatients**  **(n = 136)** | **Total**  **(n = 1092)** | **P-value** |
| Beta-blockers | 670 (70.1%) | 92 (67.6%) | 762 (69.8%) | 0.563^b^ |
| ACEi | 653 (68.3%) | 85 (62.5%) | 738(67.6%) | 0.176^b^ |
| Aldosterone | 225 (23.5%) | 46 (33.8%) | 271 (24.8%) | 0.009^b^ |
| ARNI | 0 (0.0%) | 0 (0.0%) | 0 (0.0%) | - |
| SGLT2i | 13 (1.4%) | 1 (0.7%) | 14 (1.3%) | 0.545^d^ |
| Furosemide | 730 (76.4%) | 67 (49.3%) | 797 (73.0%) | <0.001^b^ |
| Furosemide dose - mg | 40 [40, 40] | 40 [40, 40] | 40 [40, 40] | 0.631^c^ |
| Thiazide | 210 (22.0%) | 36 (26.5%) | 246 (22.5%) | 0.239^b^ |
| Antiarrhythmics | 181 (18.9%) | 22 (16.2%) | 203 (18.6%) | 0.439^b^ |
| Digoxin | 59 (6.2%) | 8 (5.9%) | 67 (6.1%) | 0.895^b^ |
| Anti platelet | 559 (58.5%) | 56 (41.2%) | 615 (56.3%) | <0.001^b^ |
| Anticoagulation | 459 (48.0%) | 58 (42.6%) | 517 (47.3%) | 0.241^b^ |
| Metformin | 250 (26.2%) | 27 (19.9%) | 277 (25.4%) | 0.114^b^ |
| Statins | 628 (65.7%) | 76 (55.9%) | 704 (64.5%) | 0.025^b^ |
| **Echo parameters** | | | | |
|  | **Inpatients**  **(n = 956)** | **Outpatients**  **(n = 136)** | **Total**  **(n = 1092)** | **P-value** |
| **LVEF - %** | 66.4 ± 2.7 | 66.7 ± 2.9 | 66.5 ± 2.8 | 0.351^a^ |
| Left ventricle end-diastolic diameter – cm | 4.5 ± 0.6 | 4.6 ± 0.5 | 4.4 ± 0.6 | 0.010^a^ |
| Interventricular septum thickness – cm | 1.2 ± 0.2 | 1.3 ± 0.4 | 1.2 ± 0.3 | 0.003^a^ |
| Left ventricle posterior wall thickness – cm | 1.1 ± 0.4 | 1.1 ± 0.9 | 1.1 ± 0.5 | 0.393^a^ |
| Left atrium volume index - ml/m² | 46.9 ± 22.5 | 44.7 ± 18.3 | 46.5 ± 21.8 | 0.480^a^ |
| Tissue doppler e/e’ ratio lateral | 16.1 ± 8.4 | 14.5 ± 6.3 | 15.9 ± 8.1 | 0.013^a^ |
| Tissue doppler e/e’ ratio septal | 20.4 ± 10.6 | 18.6 ± 8.6 | 20.1 ± 10.4 | 0.089^a^ |
| Mitral regurgitation –  moderate to severe | 138 (16.5%) | 18 (17.1%) | 156 (16.6%) | 0.873^b^ |
| Systolic pulmonary artery pressure - mmHg | 50.5 ± 17.1 | 43.8 ± 15.5 | 49.7 ± 17.1 | <0.001^a^ |
| Dilated right ventricle | 87 (29.7%) | 6 (18.2%) | 93 (28.5%) | 0.165^b^ |
| Right ventricular dysfuncation – moderate to severe | 48 (16.4%) | 3 (8.8%) | 51 (15.6%) | 0.250^b^ |
| Tricuspid regurgitation – moderate to severe | 190 (21.3%) | 17 (14.5%) | 207 (20.5%) | 0.088^b^ |

^a^- One-way ANOVA, ^b^- Chi-square test, ^c^- Kruskal-Wallis test, ^d^-Linear-by-Linear association, ^e^-calculated by CKD-EPI formula

Abbreviations: HFmrEF = heart failure with mildly reduced ejection fractions, HFpEF = heart failure with preserved ejection fraction, HFsnEF = heart failure with supranormal ejection fraction, IHD = ischemic heart disease, CKD = chronic kidney disease, COPD = chronic obstructive pulmonary disease, SBP = systolic blood pressure, CRT = cardiac resynchronization therapy, TSH = thyroid stimulating hormone, GFR = glomerular filtration rate, SGLT2i = sodium-glucose co-transporter 2 Inhibitors, ACEi = angiotensin-converting enzyme inhibitors, ARNI = Angiotensin receptor-neprilysin inhibitors, LVEF = left ventriculat ejection fraction.

# Table S4 – Possible confounders in the inpatient cohort

|  | **HFmrEF  (n = 589)** | **HFpEF (n = 3749)** | **HFsnEF (n = 956)** | **Total (n = 5294)** | **P-value** |
| --- | --- | --- | --- | --- | --- |
| Anemia during hospitalization | 95 (19.2%) | 513 (15.9%) | 118 (14.6%) | 729 (16.0%) | 0.088^a^ |
| Sepsis during hospitalization | 153 (26.0%) | 937 (25.0%) | 269 (28.1%) | 1359 (25.7%) | 0.137^a^ |
| Cirrhosis | 4 (0.7%) | 39 (1.0%) | 11 (1.2%) | 54 (1.0%) | 0.652^a^ |
| Carcinoid | 1 (0.2%) | 0 (0.0%) | 2 (0.2%) | 3 (0.1%) | 0.393^b^ |
| Gastrointestinal bleeding | 5 (0.8%) | 38 (1.0%) | 7 (0.7%) | 50 (0.9%) | 0.701^a^ |
| Echo during hospitalization | 354 (73.0%) | 2130 (67.1%) | 544 (68.3%) | 3028 (68.0%) | 0.035^a^ |

^a^- Chi-square test, ^b^- Linear-by-Linear association
